# Retrospective Evaluation of Intravenous Enoxaparin Administration in Feline Arterial Thromboembolism

**DOI:** 10.3390/ani12151977

**Published:** 2022-08-04

**Authors:** Athanasia Mitropoulou, Esther Hassdenteufel, Joanna Lin, Natali Bauer, Gabriel Wurtinger, Claudia Vollmar, Estelle Henrich, Nicolai Hildebrandt, Matthias Schneider

**Affiliations:** 1 Department of Veterinary Clinical Sciences, Small Animal Clinic, Justus-Liebig-University Giessen, 35390 Giessen, Germany; 2 Department of Veterinary Clinical Sciences, Clinical Pathophysiology and Clinical Pathology, Justus-Liebig-University Giessen, 35390 Giessen, Germany

**Keywords:** low molecular weight heparin (LMWH), enoxaparin, anti-Xa activity, clopidogrel, feline arterial thromboembolism

## Abstract

**Simple Summary:**

Feline arterial thromboembolism is a painful disease characterized by acute ischemic necrosis of one or more limbs due to cardiac diseases, hyperthyroidism, or neoplasia. Among others, medical treatment consists of preventing new thrombus formation primarily using heparin products, such as enoxaparin. This retrospective study reports clinical data, regain of perfusion, short-term outcome, and complications of 36 affected cats treated with a novel intravenous enoxaparin protocol. Furthermore, we aimed to report monitoring and management of the intravenous enoxaparin treatment for this disease. In our population, visible hemorrhage was rare. The most common causes of death/euthanasia were cardiac instability, acute kidney injury, neurological abnormalities, and limb necrosis. The hospital discharge rate was 47% overall and was significantly different between single limb (83%) and dual limb (29%) thromboembolism. Our study supports the intravenous use of enoxaparin in combination with oral clopidogrel for cats with thromboembolism as an alternative treatment method.

**Abstract:**

Induction of a hypocoagulable state is imperative in the treatment of feline arterial thromboembolism. Publications in human medicine report the use of enoxaparin intravenously in selected cases. The aim of our retrospective study was to report the regain of perfusion, short-term outcome, and complications of cats treated with a novel intravenous enoxaparin protocol (1 mg/kg bolus injection followed by 3 mg/kg/day continuous infusion) combined with oral clopidogrel administration. The secondary aim was to report the monitoring of enoxaparin with anti-Xa activity. There were 36 cats included. The probability of reaching limb reperfusion was significantly (*p* = 0.0148) higher with anti-Xa activity within or above the target range compared to results below the target range (19/21, 90% versus 11/20, 55%). The complications observed were acute kidney injury (15/36, 42%), hemorrhage (2/36, 6%), and neurological signs (6/36, 17%). The most common causes of death/euthanasia were cardiac instability, acute kidney injury, neurological abnormalities, and limb necrosis. The hospital discharge rate was 83% (10/12) for single limb and 29% (7/24) for dual limb thrombosis; the difference was significant (*p* = 0.0039). The median hospitalization time for the survivors was 119.5 (95–480) h. Our study supports the use of intravenous continuous rate infusion of enoxaparin in combination with oral clopidogrel for cats with aortic thromboembolism. We report similar discharge rates and lower hemorrhage rates than previously reported with thrombolytic treatment.

## 1. Introduction

Feline arterial thromboembolism (FATE) is a serious and painful event characterized by acute ischemic necrosis of one or more limbs, most commonly initiated by a dislodgment of thrombotic material from the left atrium [1,2]. The development of a thrombus requires an endothelial disruption, altered blood flow or blood stasis, imbalance in clotting factors, or intrinsic hypercoagulability [3,4]. The above risk factors are well known as Virchow’s triad [5]. Common underlying causes of FATE are cardiomyopathies, hyperthyroidism, and neoplasia [6,7]. The prognosis is considered guarded; therefore, more than half of the affected cats are euthanized at presentation [8]. In larger studies with cats undergoing medical therapy, the survival rate varies from 29–45% [1,2,7,8,9,10], while non-survival is associated with hypothermia [7,8,9], 2 or more limbs affected, loss of motor function (MF) [7,9], and bradycardia [7].

Possible medical treatments consist of dissolving existing thrombi with fibrinolytic agents or preventing new thrombus formation primarily using antiplatelet drugs and heparin products [11]. Fibrinolytic agents used in naturally-occurring FATE are streptokinase [9,12] (no longer available), urokinase [13], and tissue plasminogen activator (TPA) [10,14,15]. Because of the risk of reperfusion injury, these medications are currently not recommended [16]. The induction of a hypocoagulable state is imperative in treating FATE [17]. This state is achieved with the use of unfractionated heparin (UFH) [1,2,7,8,10,14], low molecular weight heparins (LMWHs) [8,9,10,18], or oral factor-Xa inhibitors [19] with or without platelet function inhibitors (acetyl salicylic acid, clopidrogrel, abciximab). In 2 large studies regarding mixed medical treatment, including UFH in all [2] or most [7] of the cats, an overall survival rate of 37% [2] and 42% [7], respectively, with relatively low risk of hemorrhage was achieved.

In general, LMWHs are considered preferable over UFH for the treatment of animals at risk of thrombosis because of their more predictable bioavailability, documented efficacy, and positive safety profile [20]. Two studies describe the subcutaneous (SQ) injection of different LMWHs in FATE (dalteparin [18], nadroparin, and enoxaparin [10]). Pharmacokinetics of SQ enoxaparin administration is defined in healthy cats [21,22,23]. In critically ill human patients, alternated kinetics for SQ medication has been demonstrated [24]. A similar phenomenon is not proven for cats but seems to be probable because many of the FATE cats present with hypothermia, hypotension, and cardiac failure. Publications in human medicine report the use of enoxaparin intravenously (IV) as a slow bolus infusion [25,26,27] or continuous rate infusion (CRI) [28,29] in special indications. Measurement of the activated factor X inhibitory (anti-Xa) activity is used to monitor the therapeutic dose of LMWH in humans, whereby a target level near 0.5–1.2 IU/mL has been used for enoxaparin CRI [28,29].

To the authors’ knowledge, there are no studies evaluating the intravenous use of enoxaparin in FATE. The primary aim of this retrospective study is to report clinical data, regain of perfusion, short-term outcome, and complications after treatment of FATE with a novel enoxaparin protocol of CRI application. The secondary aim was to report monitoring values of anti-Xa activity in regard to a specific enoxaparin-based treatment protocol for cats presenting with arterial thromboembolism (ATE).

## 2. Materials and Methods

### 2.1. Case Selection

Medical records of client-owned cats with naturally occurring FATE admitted to the university clinic from May 2013 to December 2018, initially receiving intravenous enoxaparin, were retrospectively analyzed. A diagnosis was made based on clinical observation of limb paresis or plegia and clinical evidence of decreased perfusion (lack of pulse, pale or cyanotic limb, cold, and painful limb). Additionally, the diagnosis was confirmed by a lack of identifiable blood flow at the affected limb with the Doppler technique. Owner consent to the enoxaparin treatment had to be given after a discussion with a cardiologist. Exclusion criteria were pre-treatment with heparin (UFH or LMWH), treatment with thrombolytics or oral anticoagulation drugs, and missing data.

### 2.2. Demographic, Clinical, and Diagnostic Data

Demographic data including age, sex, reproductive status, weight, and breed, history (including the time interval since onset of clinical FATE symptoms, previous treatment, and any known previous illness), physical examination findings including systolic blood pressure (SBP) measurement, radiographic findings, electrocardiographic (ECG), and echocardiographic diagnosis as well as clinicopathological data, were recorded. Concerning physical exams, panting was excluded from the statistics. A temperature below the measuring range of the thermometer was censored as 31.9 °C. Blood pressure measurements were obtained using Doppler sphygmomanometry on a peripheral limb utilizing a cuff size that was approximately 40% of the limb diameter. Thoracic radiography, ECG, and echocardiography were performed when the cat was deemed stable enough by a board-certified veterinary cardiologist or resident in a veterinary cardiology-training program. For clinically unstable cats, a focused point-of-care examination was used to identify the presence of pleural or pericardial fluid or both, the presence of B lines in the lungs, and to provide a subjective estimate of left atrial (LA) size and left ventricular (LV) systolic function. When a veterinary cardiologist was not immediately available to perform echocardiography prior to euthanasia or discharge of an affected cat, ultrasonographic images were obtained by the attending veterinarian and were subsequently reviewed by a veterinary cardiologist to confirm the presence of heart disease. Blood was collected from a peripheral or central vein into K3-ethylendiamin tetra-acetic acid (K3-EDTA) and heparinized tubes for hematological (ADVIA 2120; Siemens Healthcare GmbH, Erlangen, Germany; or ProCyte Dx hematology analyzer (IDEXX Laboratories, Westbrook, ME, USA) if presented out of hours) and biochemical (ABX Pentra C400; Horiba ABX SAS, Montpellier, France) analysis, respectively. Venous blood gas analyses (including electrolytes and lactate) were measured using a cobas b 221 POC system (Roche Diagnostics GmbH, Mannheim, Germany).

### 2.3. Treatment and Monitoring

Upon presentation, cats were stabilized in an oxygen cage, and analgesia (opioids) and potassium-free infusion were administered. After confirmation of FATE, owner consent for treatment, and admission to the intensive care unit, enoxaparin treatment was started as soon as possible.

Enoxaparin (Clexane 100 mg multidose, Sanofi-Aventis, Frankfurt am Main, Germany) was administrated as a bolus of 1 mg/kg IV followed by a CRI of 3 mg/kg/day. Monitoring of the anti-Xa activity started at the earliest 12 h after enoxaparin administration. The enoxaparin CRI was discontinued for 5 min before blood sampling in 3.13% sodium citrate tubes (Sarstedt AG & Co. KG, Nuembrecht, Germany). Anti-Xa activity was measured in citrated plasma with a commercially available chromogenic assay (Liquid anti- Xa; Diagnostica Stago S.A.S., Asnières-sur-Seine, France) using an automated analyzer (STA Compact, formerly Roche Diagnostics GmbH, Mannheim, Germany, now Diagnostica Stago S.A.S., Asnières-sur-Seine, France) directly after collection. The desired anti-Xa range (0.85–1.2 IU/mL) was defined as the upper half of the human therapeutic range [29]. In case of low anti-Xa values, the enoxaparin CRI was adjusted in 20% steps, with the exception of cats with adverse reactions (ADRs, for example bleeding from the venous catheter) observed. In case of high anti-Xa values exceeding 1.2 IU/mL and 1.8 IU/mL, the CRI dose was typically reduced by 20% and 40%, respectively.

As soon as a cat was stable enough to receive oral medication, a loading dose of clopidogrel (75 mg/cat) followed by a maintenance dose (18.75 mg/cat) the next day was administrated complementary to enoxaparin treatment. Further supportive care was tailored individually but usually consisted of passive warming, analgesia, nutrition through a nasogastric tube, and physiotherapy. Treatment for cardiac disease was included if necessary. Further interventions were implemented on a case-by-case basis. If hypotension (systolic blood pressure < 90 mmHg [10]) was present, catecholamine treatment was administrated as indicated. Dobutamine CRI (Dobutamin-ratiopharm 250 mg/50 mL, Ratiopharm, Ulm, Germany) alone or in combination with norepinephrine CRI (Arterenol, Sanofi-Aventis, Frankfurt am Main, Germany) was used. All cats were monitored with continuous ECG, clinical evaluation every 4 h, and blood pressure monitoring as often as necessary, but at least once daily. Further laboratory monitoring (blood gas analysis, hematology, and clinical biochemistry) was decided on an individual basis. The medical treatment of hyperkalemia depended on its severity and consisted of furosemide (Dimazon 50 mg/mL, Intervet, Unterschleißheim, Germany), sodium hydrogen carbonate (NaBiC 8.4%, B. Braun, Melsungen, Germany), glucose (G40, B.Braun, Melsungen, Germany) with fast acting insulin (Insuman rapid 100 I.E./mL, Sanofi, Frankfurt am Main, Germany), and stabilization of cardiac rhythm with calcium gluconate (Calciumgluconat 10%, B. Braun, Melsungen, Germany). If renal replacement therapy was deemed necessary, peritoneal dialysis (PD) was performed using a commercially available dialysis solution (Physioneal Glucose 40 1.36%, Baxter, Unterschleissheim, Germany) through an abdominal sheath-guided tube (Drainage 6 F S00124-7, Walter Veterinaer-Instrumente e. K., Baruth/Mark, Germany). After the return of circulation to the affected limb and overall clinical improvement, the enoxaparin treatment was switched from IV to SQ (1 mg/kg or 1/3 of the adjusted IV dosage, every 8 h). Cats were discharged once clinically stable with a combination of adjusted enoxaparin SQ and oral antiplatelet drugs in addition to cardiac medications.

### 2.4. Outcome Parameters and Complications

Death in the early phase (≤48 h), limb reperfusion, and survival to discharge were retrospectively evaluated. Limb reperfusion was defined as palpable pulse and/or detectable pulse signal with Doppler sphygmomanometry (Eickemeyer, Tuttlingen, Germany). The medical records of all included cats were evaluated for any complications appearing during their hospitalization. Hyperkalemia (HK) was categorized as mild (5.1–5.9 mmol/L), moderate (6.0–6.9 mmol/L), or severe (≥7.0 mmol/L) in accordance with our laboratory´s upper reference interval and similar to a previously published classification [30]. Acute kidney injury (AKI) was classified as grade 1-5 according to published guidelines [31] with modification of the border between grade 1 and 2 to the upper reference interval of our laboratory (168 µmol/L). Potassium and/or creatinine increase was classified chronologically as first potassium rise, both simultaneously or indistinguishably, or first creatinine rise. Any clinical (e.g., petechiae, hematoma, melena, bleeding from catheter entrance) or laboratory signs of blood loss (decrease of hematocrit (htc) < 0.20 L/L), including the need for blood products, were also documented.

### 2.5. Statistical Analysis

All statistical analyses were performed using commercially available statistical software (GraphPad Prism, Prism 6 for Windows, San Diego, CA, USA). Data were tested for normal distribution using the D’Agostino–Pearson omnibus test and visual inspections of the histogram. For descriptive purposes, continuous variables are reported as mean ± standard deviation or median (range), depending on the data distribution. Differences in nominal data between survivors and non-survivors were tested using a two-tailed Fisher’s exact test for categorical data. Continuous data were compared using Student’s *t*-test or the Mann–Whitney test as appropriate. For the analysis of the association between the anti-Xa value and the event of reperfusion (R) or no reperfusion (NR) (thus, time of death), in the case of multiple measurements, the measurement closest in time to the event was considered. *p*-values < 0.05 were considered significant.

## 3. Results

A total of 38 cats with confirmed FATE and treated with the described enoxaparin IV protocol were identified. Overall, 2/38 cats were excluded because of incomplete medical files.

### 3.1. Demographics and History

Of the 36 cats included, 64% (23/36) were males (20 castrated and 3 intact), and 36% (13/36) were female (all spayed). Most of the cats (31/36, 86%) were domestic shorthair cats. The remaining 5 cats were 1 of each Burmese, Himalayan, Maine Coon, Persian, and Siamese. The mean age was 9.3 ± 4.0 years. The mean weight was 5.0 ± 1.5 kg. A total of 9/36 cats (25%) had a known cardiac disease (some form of cardiomyopathy *n* = 8/9 or a heart murmur *n* = 1/9) before the thromboembolic event, and 3/9 were additionally pre-diagnosed with a second disease; 1 with diabetes mellitus, 1 with chronic kidney disease, and 1 with urinary stones (underwent surgery for removal a few days before the event). Of the 36 cats analyzed, 3 (8%) were pre-diagnosed with a non-cardiac disease, 1 each hyperthyroidism, diabetes mellitus, and feline asthma (treated with cortisone). The median time of clinical signs before presentation was 3 (1–72) h. The onset of clinical signs was reported to be associated with vomiting in 1 cat (3%).

### 3.2. Clinical Presentation

All 36 cats primarily presented mainly with loss of limb function in the affected limb(s). In 23/36 cats, both rear limbs were affected, and 1/36 cats had both rear limbs and the right front limb affected. In 12/36 cats, only 1 limb was affected (right rear limb *n* = 4/12, right forelimb *n* = 7/12, left forelimb *n* = 1/12). Weak MF was present in 7/36 cats (19%). In 2/7 cats, weak MF was observed at both rear limbs, while either the right or the left rear limb was affected in 5/7 cats. Median rectal temperature was recorded on presentation for all cats and was 37.3 (31.9–39.1) °C, whereby 24/36 (67%) of the cats were hypothermic (<37.8 °C). The median respiratory rate for 32/36 cats (4 were excluded due to panting) was 50.9 ± 14.5 breaths/min, and the mean heart rate for all 36 cats was 186.1 ± 26.1 beats/min.

### 3.3. Diagnostic Evaluations

Thoracic radiographs and echocardiography were performed on all cats. Congestive heart failure (CHF) was diagnosed in 17/36 (47%) cats. The primary cause for FATE was lung neoplasia without cardiac abnormality in 1/36 cats (3%) and cardiac disease in the rest of the cats (35/36, 97%). Diseases diagnosed by echocardiography were hypertrophic cardiomyopathy (HCM, *n* = 26) and hypertrophic obstructive cardiomyopathy (HOCM, *n* = 4), non-specific cardiomyopathy (*n* = 2), atrial myopathy (*n* = 2), and one cat had a primary bradyarrhythmia (atrial standstill) with secondary echocardiographic changes. Of the 32/36 (89%) cats in which an ECG was evaluated, the following rhythms were noted: sinus rhythm (*n* = 14), atrial fibrillation (*n* = 5), atrial premature contractions (*n* = 4), ventricular premature contractions (VPCs, *n* = 4), VPCs and atrial premature contractions (*n* = 1), supraventricular tachycardia (*n* = 1), atrial standstill and VPCs (*n* = 1), sinus bradycardia (*n* = 1), and third-degree atrioventricular block (*n* = 1).

Among the cats in which selected values of serum chemistry and electrolytes were performed at admission, commonly reported abnormalities included hyperglycemia (30/31, 97%) and high plasma concentrations of creatinine phosphokinase (CPK) (20/20, 100%). A venous blood lactate measurement was performed in 32 cats, with more than half presenting with hyperlactatemia (*n* = 20, 63%). Details on the clinical and laboratory data can be found in Table 1.

### 3.4. Anti-Xa Activity Measurement and Enoxaparin Management

**Figure 1:**
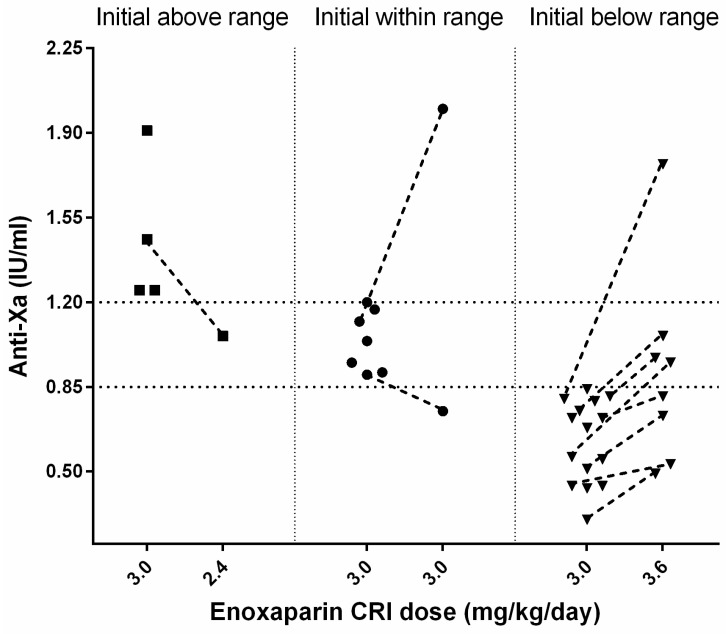
Schematical representation of anti-Xa activity measured in 26 cats with ATE during enoxaparin CRI treatment. The population is divided to initially above (square), within (circle), and below (upside-down triangle) therapeutic anti-Xa range. If available, a following measurement after appropriate dose change is depicted with a dashed line.

A total of 10 of 36 cats (28%) did not have an anti-Xa measurement because of either death (*n* = 9) after a median 27 (2–68) h of standard CRI treatment or reperfusion (*n* = 1) after 25 h. In 26/36 (72%) cats, an initial anti-Xa activity was measured during the enoxaparin standard CRI treatment with a median of 0.81 (0.3–1.91) IU/mL after 56 (14–168) h (s. Figure 1).

#### 3.4.1. Initial Anti-Xa Activity above the Therapeutic Range

A total of 4 of 26 cats had initially high median anti-Xa values of 1.36 (1.25–1.91) IU/mL after a median time of 67.5 (55–88) h; all had AKI (grade 2: *n* = 3, grade 4: *n* = 1) at the time of anti-Xa measurement. Two of four cats died (for causes unrelated to enoxaparin treatment), one cat was changed to SQ enoxaparin treatment because of reperfusion, and in one cat, the reduction of enoxaparin CRI from 3.0 to 2.6 mg/kg/day decreased the anti-Xa (from 1.46 to 1.06 IU/mL, after 71 h).

#### 3.4.2. Initial Anti-Xa Activity within the Therapeutic Range

The first anti-Xa measurement was within the target range for 7/26 cats with a median of 1.04 (0.9–1.2) IU/mL after a median time of 46 (24–68) h of enoxaparin treatment. Two of them had a re-assessment of the anti-Xa activity under the standard CRI treatment; one cat had a reduction of anti-Xa (0.75 IU/mL, within 66 h), and the other showed a marked increase (2.0 IU/mL, within 98 h) associated with grade 3 AKI.

#### 3.4.3. Initial Anti-Xa Activity below the Therapeutic Range

In 15/26 cats, initial anti-Xa values below the therapeutic range with a median of 0.68 (0.30–0.84) IU/mL were observed after a median of 48 (14–168) h of treatment. Three cats showed limb reperfusion and were further treated with increased enoxaparin SQ (1.2 mg/kg TID). In 4/15 cats, the CRI was increased from 3.0 to 3.6 mg/kg/day, but they died before re-assessment of anti-Xa activity. Overall, in 8/15 cats, a re-evaluation of anti-Xa activity after an increase of initial enoxaparin CRI from 3.0 to 3.6 mg/kg/day was performed. In all 8 cats, anti-Xa activity increased after dosage adjustment of enoxaparin. In 1/8 cats with concurrent grade 3 AKI, the anti-Xa value (1.77 IU/mL, after 72 h) exceeded the predefined target. Following enoxaparin dosage adjustment, anti-Xa activities were within the desired therapeutic range in 3/8 cats after 17, 19, and 95 h, respectively, after initiation of treatment. The remaining 4/8 cats still had anti-Xa activities below the target range after 46, 48, 49, and 60 h of treatment (s. Figure 1).

### 3.5. Anti-Xa Activity Measurement and Limb Reperfusion

**Figure 2:**
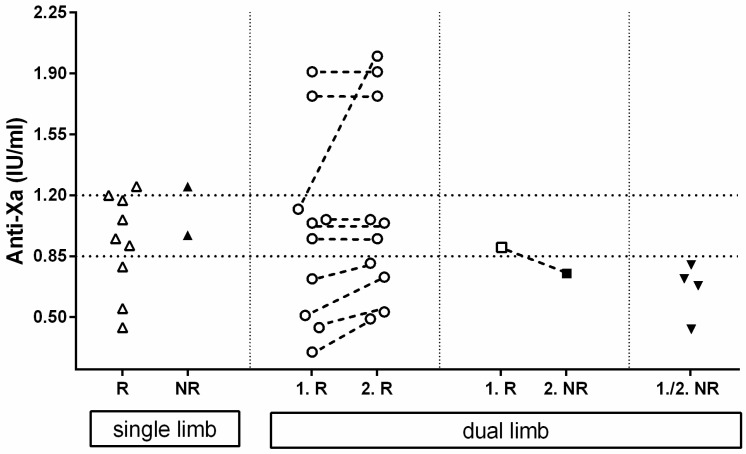
Schematical representation of anti-Xa activity of 26 cats (41 limbs) near reperfusion (R) or near death if no reperfusion (NR) occurred. Starting on the left, 11 cats with single limb ATE and reperfusion (R-open triangle) or no reperfusion (NR-closed upside triangle) and their anti-Xa values are depicted. Next are 10 cats with dual limb ATE with reperfusion (open circle) on first limb (1.R) and the second limb (2.R), and 1 cat with reperfusion on 1 limb (1.R-open square), but no reperfusion on the other limb (2.NR-closed square). Anti-Xa activities of conjugate legs are connected with a dashed line. Finally, on the right are 4 cats (8 limbs) with no reperfusion on either leg (1./2. NR-closed upside triangle).

A schematical representation of the anti-Xa activity regarding reperfusion or no-reperfusion can be found in Figure 2.

Of all the 26 cats with at least one available anti-Xa measurement during their hospitalization, 11 had single-limb ATE and 15 dual-limb ATE, leading to a total number of 41 affected limbs. A total of 9 of the 11 single-limb ATE developed reperfusion after a median enoxaparin treatment duration of 68 (36–109) h and had median anti-Xa values near the time point of reperfusion of 0.95 (0.44–1.25) IU/mL. Of these, 2 of the 11 limbs remained without circulation after 59 and 93 h of treatment, and the anti-Xa activity near the time of death was 1.25 and 0.97 IU/mL, respectively.

In 10/15 cats with a dual limb ATE, both limbs developed reperfusion. The first limb of each cat showed reperfusion after a median of 41.5 (8–230) h with a median anti-Xa activity of 1.00 (0.30–1.91) IU/mL. The second limb showed reperfusion either at the same time (*n* = 5/10) or at a later point (*n* = 5/10). The median time of reperfusion of all second limbs was 89 (27–240) h of CRI treatment, and the anti-Xa activity near reperfusion was 1.00 (0.49–2.0) IU/mL.

In 1/15 cats with dual limb ATE, reperfusion of the fist rear limb was achieved 9 h after initiation of treatment (anti-Xa activity of 0.90 IU/mL); however, the second rear limb failed to restore circulation after a total of 95 h of treatment with an anti-Xa value near the time of death of 0.75 IU/mL.

In 4/15 cats, none of the 2 rear limbs showed reperfusion after a median duration of enoxaparin CRI therapy until death of 95 (94–104) h and the median anti-Xa near the time of death was 0.70 (0.43–0.80) IU/mL.

Calculating every limb as a single event, the probability of reaching reperfusion was significantly (*p* = 0.0148) higher with anti-Xa activities within or above the target range compared to results below the target range (19/21, 90% versus 11/20, 55%).

### 3.6. Complications

#### 3.6.1. Hyperkalemia and Kidney Injury

All 36 cats had an initial potassium measurement. In 23/36 cats, creatinine was also measured (s. Table 1).

Initially, 2/36 cats had mild hyperkalemia (5.5 and 5.2 mmol/L). In the first cat, hyperkalemia resolved after fluid therapy. The second cat developed moderate hyperkalemia (6.9 mmol/L) after 5 h. Additionally, 6 cats with initially normal potassium developed moderate to severe hyperkalemia with a median of 7.45 (6.0–10.1) mmol/L after a median treatment time of 30.5 (21–106) h. Overall, in 7/36 cats, moderate to severe hyperkalemia occurred during the treatment period.

All 7 cats with moderate to severe hyperkalemia had simultaneous AKI (grade 2 *n* = 1; grade 3 *n* = 3; grade 4 *n* = 3). Out of the 7 hyperkalemic cats with AKI, 1 cat with moderate hyperkalemia was euthanized because of the underlying AKI. Management of hyperkalemia in moderate to severe cases was possible in 3/7 cats with drugs only, and in 3/7 others with peritoneal dialysis.

Additionally, 8 cats without hyperkalemia showed AKI (grade 2 *n* = 2; grade 3 *n* = 5; grade 4 *n* = 1) during treatment. The cat with grade 4 AKI was treated with peritoneal dialysis.

Overall, 15/31 (48%) cats with available consecutive creatinine data had AKI; this was less common but not significantly different (*p* = 0.0659) in single limb (3/12, 25%) than in dual or more limb (12/19, 63%) FATE. The development of grade 2 or higher AKI was significantly (*p* = 0.032) associated with non-survival.

#### 3.6.2. Blood Loss and Anemia

In 2/36 cats, a visible hemorrhage was documented. One cat developed hematuria after placement of a urinary catheter, and the other cat had epistaxis after placement of nasoesophageal tube. None of the 36 cats showed clinical signs of acute blood loss. Twenty-eight cats had at least one re-evaluation of the htc during their hospitalization time. In these cats, there was a significant (*p* < 0.0001) reduction in htc from 0.40 ± 0.06 to 0.26 ± 0.07 over a median time of 84 (15–325) h. A total of 6 cats, including the 2 cats with visible hemorrhage, had the lowest hematocrit, less than 0.20 L/L with a median of 0.18 (0.13–0.19) L/L at a median time of 95 (43–325) h. A total of 2 cats received a blood transfusion: 1 with the epistaxis (htc 0.19 L/L at 214 h) and 1 with extremely low htc (0.13 L/L at 325 h). In none of the anemic cats, the anemia was the cause of death, but 5/6 cats were euthanized because of their underlying disease. The clinically relevant anemia was not related to non-survival (*p* = 0.182).

#### 3.6.3. Neurological Signs

A total of 6 of 36 cats (1 front limb and 5 both rear limbs) developed neurological signs (17%). Those included generalized seizures (*n* = 3), coma (*n* = 2), and loss of cranial nerve function (*n* = 1) after a median of 48 (12–208) h of enoxaparin treatment. All cats were finally euthanized. The development of neurologic signs did not reach significance for non-survival (*p* = 0.0656).

### 3.7. Outcome

A total of 20 out of the 36 cats showed reperfusion of all affected limbs (56%), and 3 of these died before discharge; therefore, the survival to discharge was 17/36 (47%) after a hospitalization length of 166 (95–480) h. A total of 10 out of the 12 (83%) cats with single limb FATE were discharged after a median hospitalization time of 156 (134–264) h. Of cats with dual limb thromboembolism, 7/24 (29%) survived to discharge after a median hospitalization time of 288 (95–480) h; the difference in discharge rate was significant (*p* = 0.0039).

Early death (≤48 h) occurred in 8/24 (33%) cats with at least 2 limbs affected and in none of the single limb thromboembolism cases. A total of 11 more cats died after the first 48 h. The cause of death was euthanasia due to the owner’s (emotional or financial) decision for 2 cats after 5 and 26 h of treatment. Additionally, 2 cats showed sudden death caused by ventricular fibrillation (after 8 and 35 h of treatment), and 3 cats were euthanatized because of progression of CHF (after 4, 60, and 96 h of treatment). A further 3 cats were euthanized because of neurological abnormalities (after 20, 72, and 95 h of treatment) and 5 cats because of AKI after a median of 70 (45–104) h of treatment. Finally, in 4 cats, the cause of euthanasia was limb necrosis after a median time of 196.5 (95–332) h of treatment.

## 4. Discussion

The results of this retrospective study indicate that intravenous CRI enoxaparin is a safe option for the treatment of FATE. In 26/36 cats with available anti-Xa measurement, limb reperfusion (30/41, 73%) was associated with higher anti-Xa activity. The present study population’s characteristics were similar to previously published studies evaluating FATE [2,7,8,9,10]. Overall survival to discharge was 47%. This observation is consistent with previous studies in cats treating FATE with SQ UFH/LMWH or fibrinolytic agents in combination with platelet inhibitors. Specifically, administration of SQ UFH with aspirin (37% [2] and 42% [7]), LMWH in combination with clopidogrel/aspirin (29%) [10], streptokinase (33%) [9] or TPA with UFH (27%) [15] or with LMWH and antiplatelet agents (44%) [10]. Survival was significantly higher in single limb (83%) compared to dual (or more) limb (29%) FATE. This has already been described in other cat studies [7,9]. In humans, the 30-day survival rate for acute aortic (dual limb) occlusion is comparably low (24%) [32].

AKI was a common problem for single limb (25%) and dual limb (63%) FATE. The prevalence of AKI in FATE (57%) [2] and in humans with distal aortic occlusion is reported to be similarly high (52%) [32]. However, another study investigating dual limb FATE described a lower prevalence of AKI (28%) [10]. A possible explanation for this discrepancy is the difference in the definition of AKI. A second cause is that the mentioned study [10] counted cats with early hyperkalemia followed by increased creatinine as reperfusion injury and not as AKI; this was not done in our study. In some human patients, the aortic occlusion also included the renal arteries [32], which could also be relevant in cats with ATE and AKI. This was not examined in detail in the present study or in any of the previously mentioned cat studies [2,10]. Furthermore, in humans, rhabdomyolysis with myoglobinemia, hyperkalemia, and lactic acidosis, often accompanied by hypotension, has been reported to cause AKI in 51% of patients [33]. Except for one cat with urinary bleeding, none of the cats evaluated had obviously discolored urine, but all cats examined had high CPK, and some had refractory hypotension. Therefore, it is possible that in some cats, AKI was caused by rhabdomyolysis. Moreover, hypoxemia or hypotension associated with the underlying cardiac disease and the combination of diuretic treatment could have caused AKI.

In the present study, 17% of the cats developed neurological signs, which is markedly lower than reported previously in a study in cats with FATE using TPA as a thrombolytic agent (45%) [15]. In humans, TPA has shown a risk of intracranial hemorrhage [34]. In the present study, further diagnostic evaluation or necropsy was not performed, and an intracranial hemorrhage cannot be ruled out. Other possible reasons mentioned in the literature include micro-thrombosis of the brain vasculature [15], use of vasopressors [35], or generally poor perfusion of the brain [36].

This study is the first to report treatment of FATE with a CRI of enoxaparin. A possible rationale for using enoxaparin CRI in FATE is the need for a constant level of anticoagulation during the treatment of unstable cats. Similarly, some clinicians prefer IV administration over SQ for critically ill patients to obtain a more constant, predictable drug action in a population with variable pharmacokinetics, as reported for many drugs [37]. In human medicine, a number of reports describing the use of enoxaparin CRI are published for preventing thromboembolic events during hemodialysis [38], treatment of pulmonary thromboembolism [39,40], or in special patient populations [41,42]. Moreover, in critically ill humans, it is demonstrated that alternated kinetics for SQ LMWH occurs [24] and that vasopressors interfere with this treatment [43]. Although studies in critically ill cats are missing, it seems probable to be also true as many of the FATE cats are presented with hypothermia, hypotension, cardiac failure, and need for treatment with vasopressors. The safety of enoxaparin with this atypical method of administration was assessed based on the most common ADRs, namely hemorrhage reported in cats with SQ administrated enoxaparin or LMWHs in general [18,21,44]. In the present study, a visible hemorrhage was detected in only 2/36 (6%) cats. Both cats had undergone a procedure (nasoesophageal tube or urine catheter placement) while receiving enoxaparin CRI, which may have caused irritation of the mucosa and onset of hemorrhage. Moreover, the 2 cats with hemorrhage, and 3 additional cats (5/36, 14%) developed an anemia severe enough to require a blood transfusion. There are numerous explanations for the observed complication. Repeated blood withdrawals necessitating blood transfusions is common in critically ill cats [45] and is possibly the reason for the occurrence of severe anemia in the present population, although it cannot be safely decided retrospectively. Other causes for development of anemia include gastrointestinal bleeding such as in humans with AKI [46], enoxaparin use in AKI [28], hemolysis, and systemic inflammatory response syndrome.

The present protocol with bolus IV followed by CRI was adopted from an experimental study in dogs for treating induced coronary thrombosis [47]. Both the starting IV dose (1 mg/kg) and the initial CRI dose (3 mg/kg/day) of enoxaparin were extrapolated from previously published SQ kinetics in healthy cats [23]. The selected CRI dose was relatively high compared to the adult human CRI dose (2 mg/kg/day) [28,29]. However, in infants, the need for higher enoxaparin doses is described [26,48]. In addition, two studies [21,44] reported faster kinetics of enoxaparin in cats compared to humans.

Anti-Xa levels have shown to be a poor predictor for enoxaparin antithrombotic effect in an experimental cat study [22]. Additionally, anti-Xa target levels are not yet established in feline medicine [49] and are sometimes considered to be non-essential [22]. In contrast, in human medicine, it is proven that considerable variations in anti-Xa clearance occur between different patient populations, and distinct anti-Xa target levels are advised [28]. For people, published anti-Xa activity targets include enoxaparin in prophylactic SQ administration (0.1/0.3–0.5 IU/mL) [26,27], therapeutic SQ or IV slow bolus infusion (0.5/0.6–1.0 IU/mL) [26,27,48], and therapeutic CRI (0.5–1.1/1.2 IU/mL) [28,29]. In feline medicine, the anti-Xa therapeutic range for cats has been taken from human medicine (0.5–1.0 IU/mL) [21,23]. The therapeutic target for the protocol used here was set within the upper half of the human therapeutic CRI range and was higher than in healthy cats to achieve a better effect without risking the patient.

Impaired kidney function is a well-known factor for reduced LMWH clearance in humans [28,29]. This seems to be true also for cats, as in the present study, anti-Xa values above the target range were associated in every case with AKI. The selected anti-Xa target range was not achieved in 58% of the cat population, and 15% (all cats with AKI) had higher anti-Xa activities than targeted. According to these results and similar to human medicine [29], the current enoxaparin CRI protocol for FATE treatment might be adjusted in the future. Starting CRI dose in cats with impaired renal function should be lower and in those with normal kidney function higher than in the current protocol. As proposed in children [27], the dose adjustment could be made in shorter intervals (every 24–48 h).

This study has several limitations. The main limitation is its retrospective nature resulting in a lack of standardization of clinical and laboratory re-assessment. Some data were missing in individual cats, and due to the severity of the symptoms, some cats did not have a full laboratory examination at presentation, or no re-checks were performed before death. We used a definition for AKI from the literature with an adapted cutoff suitable to our institution’s laboratory. In some cases, it was impossible to investigate if these cats already had a pre-existing chronic kidney injury. Moreover, it is possible that visible but mild hemorrhages were missed from the records. The group size of our study is small, a control group is missing, and anti-Xa concentration was not measured at standardized time points. Moreover, all cats received oral antiplatelet drug (clopidogrel) as soon as oral medication was possible. Finally, the outcome was influenced by treatment cost and generally by client decisions.

## 5. Conclusions

The data of this retrospective study support the use of enoxaparin CRI in combination with oral clopidogrel for FATE. The CRI protocol is safe, especially in non-azotemic cats. Limb reperfusion was positively correlated with higher anti-Xa activity. The true benefit of the reported protocol over treatment with UFH or thrombolytics remains to be evaluated in properly designed future prospective studies.

## Figures and Tables

**Table 1:** Data from the 36 cats presenting with arterial thromboembolism.

Parameter	Data ^a^	N ^b^	Percentage (%)	Reference Range
***Signalment***				
Gender Male:Female	23:13	36	n/a *	n/a
Age (years)	9.3 ± 4.0	36	n/a	n/a
Weight (kg)	5.0 ± 1.5	36	n/a	n/a
***Clinical signs***				
Duration of illness (h)	3 (1–72)	36	n/a	n/a
Limb(s) affected:		36		
− 1 front limb	23		64%	n/a
− both rear limbs and one front	1		3%	n/a
− 1 rear limb	4		11%	n/a
− both rear limbs	8		22%	n/a
Weak motor function present	7	36	19%	
Body temperature (°C)	37.3 (31.9–39.1)	36	n/a	37.8–39.2
Respiratory rate (breaths/min)	50.9 ± 14.5	32	n/a	12–38
Heart rate (beats/min)	186.1 ± 26.1	36	n/a	150–240
CHF * at presentation	17	36	47%	
***Selected laboratory data***				
Potassium (mmol/L)	3.94 ± 0.60	36	n/a	3.6–5.0
Creatinine (µmol/L)	115 (62–388)	23	n/a	<168
Phosphate (mmol/L)	1.61 (0.90–3.22)	22	n/a	0.8–1.9
Htc (L/L)	0.40 ± 0.06	36	n/a	0.24–0.45
Creatine phosphokinase (IU/L)	29,975 (420–400,000)	22	n/a	<205

^a^ mean ± standard deviation is reported for normally distributed continuous data; median (range) is reported for non-normally distributed continuous data. ^b^ Number of cases for which data were reported. * CHF: congestive heart failure, Htc: Hematocrit value, n/a: non-applicable.

## Data Availability

The datasets used and/or analyzed during the current study are available from the corresponding author upon reasonable request.
